# The Importance of Team Health Climate for Health-Related Outcomes of White-Collar Workers

**DOI:** 10.3389/fpsyg.2017.00074

**Published:** 2017-01-30

**Authors:** Heiko Schulz, Hannes Zacher, Sonia Lippke

**Affiliations:** ^1^Institute of Psychology, University of LeipzigLeipzig, Germany; ^2^Department of Brand and Marketing Management, Market and Marketing Analysis, Techniker KrankenkasseHamburg, Germany; ^3^School of Management, University of QueenslandBrisbane, QLD, Australia; ^4^Department of Psychology and Methods, Health Psychology and Behavioral Medicine, Jacobs University BremenBremen, Germany

**Keywords:** organizational climate, health, presenteeism, teams, work ability

## Abstract

Occupational health researchers and practitioners have mainly focused on the individual and organizational levels, whereas the team level has been largely neglected. In this study, we define *team health climate* as employees’ shared perceptions of the extent to which their team is concerned, cares, and communicates about health issues. Based on climate, signaling, and social exchange theories, we examined a multilevel model of team health climate and its relationships with five well-established health-related outcomes (i.e., subjective general health, psychosomatic complaints, mental health, work ability, and presenteeism). Results of multilevel analyses of data provided by 6,449 employees in 621 teams of a large organization showed that team health climate is positively related to subjective general health, mental health, and work ability, and negatively related to presenteeism, above and beyond the effects of team size, age, job tenure, job demands, job control, and employees’ individual perceptions of health climate. Moreover, additional analyses showed that a positive team health climate buffered the negative relationship between employee age and work ability. Implications for future research on team health climate and suggestions for occupational health interventions in teams are discussed.

## Introduction

Based on theorizing on organizational climate (Schneider et al., 2013), signaling theory (Connelly et al., 2011), and social exchange theory (Blau, 1964), the goal of this study is to examine relationships between team health climate and important health-related outcomes of white-collar workers. While *psychological climate* is defined as employees’ individual perceptions of their work environment (including policies, practices, and procedures), *team and organizational climates* refer to employees’ shared perceptions of their work environment within these respective units (Schneider and Reichers, 1983; Glick, 1985). A large body of research has shown that specific psychological, team, and organizational climates are associated with relevant outcomes (James et al., 2008; Kuenzi and Schminke, 2009; Schneider et al., 2013). For instance, studies have demonstrated effects of team and organizational safety climates on safety knowledge, motivation, attitudes, and performance (Griffin and Neal, 2000; Zohar, 2000; Probst, 2004; Clarke, 2006; Dollard and Bakker, 2010).

Consistent with previous work on health climates in the occupational health psychology literature (Ribisl and Reischl, 1993; Basen-Engquist et al., 1998; Sonnentag and Pundt, 2013, 2016; Zweber et al., 2016), we define *team health climate* as employees’ shared perceptions of the extent to which their team is concerned, cares, and communicates about health issues. In line with Morgeson and Hofmann (1999), we assume that team health climate emerges through a bottom-up process during which employee interactions form a collective construct at the team level that, in turn, impacts on health-related outcomes. We argue that team health climate is positively related to favorable health-related outcomes because employees are motivated to behave consistent with expectations and common practices in their teams (i.e., team climate; Ehrhart and Naumann, 2004; Schneider et al., 2013). Moreover, signaling theory (Ostroff and Bowen, 2000; Connelly et al., 2011) suggests that a positive team health climate signals to employees that their health is valued. Social exchange theory (Blau, 1964; Tetrick and Peiró, 2016) proposes that employees are more committed to maintaining and improving their health when they perceive that their health is valued by others.

Despite an increased interest among researchers and practitioners in understanding how psychosocial work characteristics impact on health-related outcomes (Sparks et al., 2001; Parker, 2014), so far very little empirical research on relationships between team health climate and relevant outcomes exists. This is surprising, given that organizational psychologists have early emphasized the potential importance of teams and health climate for employee health (Ilgen, 1990; Stokols, 1992; Sonnentag et al., 1994). The World Health Organization (1948) defines health as “a state of complete physical, mental, and social well-being and not merely the absence of disease and infirmity” (note that well-being is a general term that can refer to various positive conditions of individuals and groups).

It is important to study health-related outcomes and their predictors in the work context because health is an essential prerequisite for labor force participation and employee contributions in the workplace; poor health is associated with lower quality of life, lower productivity, and absenteeism at the individual level and immense costs due to productivity losses and health care expenditures at the organizational and societal levels (Danna and Griffin, 1999; Cartwright and Cooper, 2013). In the year 2014, on average, a white-collar worker in Germany was 12 days on sick leave, and there were 122 instances of sickness absence per 100 white-collar workers who are members of public health insurances – these statistics are very similar to the averages across different occupations (Bundesanstalt für Arbeitsschutz und Arbeitsmedizin, 2016). The most common reasons for sickness absences were musculoskeletal and mental health problems. The total costs of the inability to work due to sickness in Germany are estimated at 57 billion Euro based on average worker salary, and at 90 billion Euro based on lost productivity (i.e., workers’ inability to create value through their work when they are on sick leave; Bundesanstalt für Arbeitsschutz und Arbeitsmedizin, 2016).

As health-related outcomes, we investigate employees’ general subjective health, psychosomatic complaints, mental health, work ability, and presenteeism in this study. These frequently investigated constructs broadly represent the domains of physical health (i.e., general subjective health and psychosomatic complaints; individuals typically think of their physical health when asked about their general health; Ware et al., 1996), mental health, and behavioral indicators of health (work ability and presenteeism; Ng and Feldman, 2013). Our research is important for individual employees, organizations, and society as a whole, as improving the health climate in teams might benefit employee health and, in turn, improve productivity and reduce absenteeism and health care costs.

### Previous Research on Team Health Climate

A number of previous studies examined relationships between health climate perceptions and outcomes at the individual employee level. Using a sample of 203 employees from seven companies, an early study by Ribisl and Reischl (1993) found that health climate perceptions were negatively related to self-reported symptoms of physical ill-health (e.g., headache, poor appetite, dizziness), and positively related to a range of beneficial self-reported health behaviors (i.e., exercise, nutrition, and reduced smoking) and job attitudes (i.e., job satisfaction and low strain). A study by Basen-Engquist et al. (1998) investigated health climate perceptions of 6,867 employees from 40 worksites. In contrast to the study by Ribisl and Reischl (1993), these researchers reported that health climate perceptions aggregated to the worksite level did not significantly correlate with employee health behaviors such as healthy eating and smoking.

Furthermore, results by Ernsting et al. (2013) showed that health climate at baseline positively predicted affective commitment at follow-up: those who perceived a positive health climate showed higher levels of affective commitment 5 months later. Sonnentag and Pundt (2016) defined organizational health behavior climate as “employee perceptions of organizational efforts to promote health behavior” (p. 260). In three studies, these authors developed and validated scales to assess two dimensions of organizational health behavior climate (i.e., healthy eating, physical exercise). They showed that organizational health behavior climate was positively associated with healthy eating, exercise identity, and negatively associated with body mass index. Finally, Zweber et al. (2016) developed a three-dimensional scale (with foci on workgroup, supervisor, and organization) to assess workplace health climate from the perspective of employees. Using individual-level data, they found that the measure was positively related to employee health, and negatively related to job stress and fatigue.

Overall, previous research showed that perceptions of health climate are associated with health-related outcomes at the individual level. However, these findings are not conclusive regarding outcomes of health climate conceptualized at higher levels, because studies either did not assess health-related outcomes, or because the number of units at the team or organizational level – if examined at all – was rather small, agreement among employees in health climate perceptions was not reported, or data were not analyzed using multilevel methods that simultaneously account for within- and between-unit variance in employees’ health climate perceptions. Thus, it remains unknown whether a positive and shared team health climate is associated with more favorable health-related outcomes among employees.

### Health-Related Outcomes

Before developing our hypotheses, in this section we define and explain the five health-related outcomes that we examined in relation to team health climate in the current study. We chose these health-related outcomes because (a) they have important implications for individuals, organizations, and society, (b) they are frequently investigated in the occupational health psychology literature, and (c) because they represent three important health domains, that is, physical health (i.e., general subjective health and psychosomatic complaint), mental health, and behavioral indicators of health (work ability and presenteeism; Ng and Feldman, 2013).

*Subjective general health* is an overall assessment of one’s current health status (Kristensen et al., 2005). Research has shown that subjective general health is positively correlated with indicators of objective physical health, particularly symptom checklists and results of medical examinations based on strict protocols (Pinquart, 2001). Moreover, subjective general health has been found to negatively predict mortality; individuals with greater subjective general health tend to live longer (DeSalvo et al., 2006a).

*Psychosomatic complaints* involve employee perceptions of physical symptoms that may also have a psychological cause, such as headaches, back, neck, and shoulder pain, and concentration difficulties (Frese, 1985). Research has shown that psychosomatic complaints lead to increased absenteeism from work (De Boer et al., 2002).

*Mental health* is defined as a psychological syndrome composed of positive feelings and positive functioning in different life domains (Keyes, 2002). Employee mental health has been shown to be positively related to subjective and objective measures of job performance (Wright et al., 1993; Bond and Bunce, 2003; Zacher et al., 2012) and company productivity (Goetzel et al., 2004).

*Work ability* refers to employees’ assessment of the extent to which they possess the physical and mental capabilities to meet their work demands (Ilmarinen et al., 1997; McGonagle et al., 2015). Research has demonstrated that work ability is positively associated with employees’ retirement age (Sell, 2009), quality of life before and after retirement (Ilmarinen, 2009), and disability status (Alavinia et al., 2009).

Finally, *presenteeism* means that employees go to work despite feeling they should have taken sick leave due to their perceived health status (Aronsson et al., 2000). Presenteeism is associated with negative individual, organizational, and societal outcomes, such as deteriorating employee health over time and productivity losses (Johns, 2010), as well as high health care and insurance costs for employers (Goetzel et al., 2004).

### Hypothesis Development

The organizational and team climate literature shows that specific workplace climates (e.g., safety climate) are most strongly related to relevant and specific employee outcomes (e.g., safety performance; Patterson et al., 2005; González-Romá et al., 2009; Schneider et al., 2013). In other words, predictive validity of climate measures is highest when the focus of the climate construct matches with the nature of the outcomes. Based on climate theory (Schneider et al., 2013), we therefore expect that team *health* climate is associated with five *health*-related outcomes. Specifically, we expect that team health climate is positively related to employees’ subjective general health, mental health, and work ability, and negatively related to psychosomatic complaints and presenteeism, above and beyond employees’ idiosyncratic perceptions of team health climate (i.e., psychological team health climate).

Climate involves “the shared perception of the way things are around here” (Reichers and Schneider, 1990, p. 22). Climate theory suggests that, due to social norms and expectations, employees are motivated to behave consistent with common practices in their team (Ehrhart and Naumann, 2004). Moreover, a positive team health climate indicates that knowledge, skills, and support for maintaining health and healthy behaviors exist in the team which, in turn, should enhance health communication and outcomes among team members (Sonnentag and Pundt, 2016).

Relationships between team health climate and health-related outcomes can be further explained by signaling theory (Ostroff and Bowen, 2000; Connelly et al., 2011) and social exchange theory (Blau, 1964; Tetrick and Peiró, 2016). According to signaling theory, a positive team health climate signals to employees that the topic of health is valued in their team and, accordingly, that employees are expected to take care of their own health and support others in this regard as well (Connelly et al., 2011). These expectations are likely to motivate employees to maintain and improve their health. Social exchange theory further suggests that employees who perceive that their health is valued by the team become more committed to their team and its goals and, therefore, invest increased effort to maintain and improve their health (Blau, 1964).

Overall, if a team is very concerned, cares, and communicates about health issues, and team members are expected to take care of their own health and support others in maintaining and improving their health, this should result in favorable health-related outcomes among employees (i.e., increased subjective general health, mental health, and work ability, and reduced psychosomatic complaints and presenteeism). Contrarily, if health is not a priority in the team and members are not expected by others in the team to take care of their own health and support others in this regard, this should result in less favorable health-related outcomes. Thus, based on climate theory (Schneider et al., 2013), signaling theory (Connelly et al., 2011), and social exchange theory (Blau, 1964), we propose that team health climate is a work-related resource (Hobfoll, 2011) that resides at the team level and benefits employees’ health-related outcomes.

Accordingly, we examine a multilevel model of team health climate and its relationships with five well-established health-related outcomes (i.e., subjective general health, psychosomatic complaints, mental health, work ability, and presenteeism) and test the following hypotheses:

*Hypothesis 1:* Team health climate is positively related to subjective general health.
*Hypothesis 2:* Team health climate is positively related to mental health.
*Hypothesis 3:* Team health climate is positively related to and work ability.
*Hypothesis 4:* Team health climate is negatively related to psychosomatic complaints.
*Hypothesis 5:* Team health climate is negatively related to presenteeism.

## Materials and Methods

### Participants and Procedure

Data for this study came from 6,449 white-collar workers working in 621 teams of a large statutory health insurance organization in Germany. Each participant could be unequivocally linked to one work team because each individualized online survey link was connected to a specific team code. All procedures performed in this study were in accordance with the ethical standards of the 1964 Helsinki Declaration and its later amendments or comparable ethical standards. Because this study was carried out in an occupational setting and approval was given by the work council (which, in the business context, is comparable to a university ethical committee) including a confidentially note, no university ethical approval was required.

Of the employees, 1.9% were under 20 years old, 24% were between 21 and 30 years, 21.3% were between 31 and 40 years, 34.4% were between 41 and 50 years, 17.4% were between 51 and 60 years, and 1% was older than 60 years. In terms of job tenure, 24.8% had worked for less than 5 years in the organization, 28.5% between 6 and 15 years, and 46.7 more than 15 years. The teams were distributed across the country and are responsible for tasks such as arrangements of ambulatory and hospital care, membership administration, marketing and sales, and customer relationships.

Employees’ voluntary participation in an online questionnaire during work hours was encouraged by the organization’s management via a letter in the intranet and by a note from the human resource department included with the pay slips. The questionnaire and scales were kept relatively short to reduce participant attrition and time investment. Based on requests of the organization’s management, staff council, and department of data protection, gender of employees was not measured in the questionnaire.

At the time of the study, approximately 11,000 individuals worked for the organization and 8,070 of them completed at least one item in the online questionnaire (73%). We excluded responses from team leaders (*n* = 680), participants who could not be allocated to a specific team (*n* = 470), participants with missing data in the study variables (*n* = 458), and participants from teams with less than two members (*n* = 13). We excluded team leaders because they are not only part of the team they are leading, but also part of a leadership team at a higher organizational level, which could have resulted in biased responses to the team health climate items. The number of participants per team (i.e., team size) ranged from two to 31 employees (*M* = 10.38, *SD* = 5.10) and the average response rate at the team level was 72.44% (range from 40.54 to 100%).

### Measures

#### Team Health Climate

We assessed team health climate with three items adapted from a short, reliable, and well-validated general organizational health climate scale developed by Sonnentag and Pundt (2013). We used the procedure outlined by Brislin (1970) to translate the items from English into German. Consistent with our definition of team health climate, the items reflect whether members of a team are concerned, care, and communicate about health: “The topic of health is present in our team meetings and other team events,” “In our team, it is expected that one takes care of his/her health,” and “In our team we exchange ideas about healthy living” (the original items by Sonnentag and Pundt, 2013, were “Here, one’s attention is drawn to health issues during presentations and other events,” “Here, most people expect that one takes care of one’s health,” and “Here one exchanges ideas about how to live healthy”).

The items used a referent-shift approach in that the employees rated their team and not their own attitudes (Chan, 1998). Employees responded using a 4-point scale ranging from 1 (*disagree*) to 4 (*agree*). To compute psychological team health climate at the individual level (i.e., individuals’ perceptions of the health climate in their team), we averaged scores across items for each employee. Cronbach’s alpha for this scale was 0.71 in the current study. For our measure of team health climate at the team level, we aggregated employee responses to the team level. This was justified by a significant intraclass correlation coefficient (ICC[1]) of 0.20 (*p* < 0.001), indicating that 20% of the total variance resided at the between-group level, as well as an ICC(2) of 0.73, indicating satisfactory reliability of the team means (Bliese, 2000).

At the time this study was conducted, recently published short scales to measure team health climate were not yet available (Sonnentag and Pundt, 2016; Zweber et al., 2016), and existing scales did not explicitly focus on teams (e.g., they also include questions about supervisors; Basen-Engquist et al., 1998). Therefore, we conducted a pilot study using Amazon’s Mechanical Turk platform to gather validity evidence for our team health climate scale. We asked 150 workers to respond to our three items as well as five items developed by Basen-Engquist et al. (1998) to measure health climate (i.e., “At my workplace, sometimes we talk with each other about improving our health and preventing disease,” “Most employees here are very health conscious,” “Around here they look at how well you take care of your health when they consider you for promotion,” “My supervisor encourages me to make changes to improve my health,” and “Supervisors always enforce health-related rules (smoking policies, requirements about medical examinations, etc.).” The correlation was positive and strong, *r* = 0.78 (*p* < 0.001), providing evidence for the convergent validity of our three-item team health climate measure.

#### Subjective General Health

We measured subjective general health with a single item (“How would you describe your current health?”) adapted from Kristensen et al. (2005). Employees provided their answer on a 5-point scale ranging from 1 (*poor*) to 5 (*very good*). The original item is “Would you say your health is excellent, very good, fair, or poor?” We translated and back-translated the item from English into German to ensure similarity with the original wording (Brislin, 1970). Research has demonstrated good reliability and validity of single-item subjective general health measures (Lundberg and Manderbacka, 1996); for instance, the item has been found to negatively predict mortality (DeSalvo et al., 2006a,b).

#### Psychosomatic Complaints

We measured psychosomatic complaints with a German short version of the complaint list developed by Fahrenberg (1975), which is a frequently used scale in German-speaking countries (e.g., Frese, 1999; Zacher and Schulz, 2015). It is similar to a well-established English-language scale of psychosomatic complaints (Caplan et al., 1975). The six items describe relevant symptoms for a sample of white-collar workers (“How often do you experience the following strains during or immediately after work?”): headaches, backaches, tiredness, neck pain, shoulder pain, and difficulties concentrating. The items were answered on a 5-point scale ranging from 1 (*never*) to 5 (*almost daily*). In the current study, Cronbach’s alpha for the scale was 0.81.

#### Mental Health

We assessed mental health with two screening items in German language that are frequently used in clinical assessments and that have been well-validated in previous research (Kroenke et al., 2003; DGPPN et al., 2009). The items are “In the past 4 weeks, did you often feel down, depressed or hopeless?” and “In the past 4 week, did you have little interest or pleasure in doing things that you usually like to do?” Employees responded with either *no* (1) or *yes* (2). Due to the ordinal nature of both items, we computed the Spearman rank-order correlation coefficient (*r*_s_ = 0.67) as an estimate of reliability. As this estimate was based on two items only, we deemed the coefficient to indicate acceptable reliability.

#### Work Ability

We assessed employees’ perceptions of their work ability with two items from the German version of the work ability index (Tuomi et al., 1997; WAI-Netzwerk, 2015): “How do you rate your current work ability with respect to the physical demands of your work?” and “How do you rate your current work ability with respect to the mental demands of your work?” Previous research has demonstrated good reliability and convergent validity of this two-item measure (Ahlstrom et al., 2010). The response format was a 5-point scale ranging from 1 (*very poor*) to 5 (*very good*). We averaged the items to form a single work ability score (note that additional analyses for each item yielded very similar results to the ones reported in the “Results” section). In the current study, Cronbach’s alpha was 0.71, which is satisfactory for a two-item measure.

#### Presenteeism

We measured presenteeism with a single item adapted from Aronsson et al. (2000) and Demerouti et al. (2009): “Did you go to work in the past 12 months, even though you were sick or felt sick?” Employees responded on a 4-point scale ranging from 1 (*no, never*) to 4 (*yes, more than five times*). The original item by Aronsson et al. (2000) is “Has it happened over the previous 12 months that you have gone to work despite feeling that you really should have taken sick leave due to your state of health?” We used the procedure outlined by Brislin (1970) to translate the item from English into German. Previous research has successfully utilized this single-item measure, showing for instance that job demands predicted presenteeism ratings (Demerouti et al., 2009).

#### Demographic and control variables

Based on requests of the organization’s management, staff council, and department of data protection, gender of employees was not measured in the questionnaire, and age and job tenure were assessed using several bands. Specifically, age was coded 1 = *20 years or younger*, 2 = *21–30 years*, 3 = *31–40 years*, 4 = *41–50 years*, 5 = *51–60 years*, and 6 = *older than 60 years*, and job tenure was coded 1 = *5 years or less*, 2 = *6–15 years*, and 3 = *16 years or more.* We controlled for age and job tenure, because research suggests that these time-related constructs are associated with health-related outcomes (Maertens et al., 2012; Stephan et al., 2012; Ng and Feldman, 2013). We controlled for number of participants from each team as a proxy for team size, because some research suggests that team size is negatively related to positive team climate due to lower average individual participation in larger teams (Colquitt et al., 2002).

Moreover, we measured and controlled for job demands (i.e., perceived stressors in the work environment) and job control (i.e., the perceived amount of autonomy and decision latitude an employee has with regard to work responsibilities). The job demands-control model (Karasek, 1979) and empirical research on this model suggest that these job characteristics are related to health-related outcomes (van der Doef and Maes, 1999; de Lange et al., 2003). Specifically, job demands should relate negatively to favorable health-related outcomes, whereas job control should relate positively to favorable health-related outcomes.

Job demands were measured with a reliable and well-validated German version of the five-item effort scale from the effort-reward imbalance questionnaire (Pfaff et al., 2004; Siegrist et al., 2004). The effort scale is a suitable indicator of job demands, as noted by Siegrist et al. (2004): “Effort is measured by five or six items that refer to demanding aspects of the work environment (three items measuring quantitative load, one item measuring qualitative load, one item measuring increase in total load over time)” (p. 1486). An example item is “I have constant time pressure due to a heavy work load.” We did not include the sixth item measuring physical load because Siegrist et al. (2004) suggested that “…the five-item version excluding physical load has been found to be psychometrically appropriate in samples characterized predominantly by white-collar jobs” (pp. 1486–1487). The 5-point response scale ranged from 1 (*no – does not apply*) to 5 (*yes – does apply and I feel very distressed about this*). In the current study, Cronbach’s alpha for the scale was 0.77.

Job control was measured with four items from a reliable and well-validated German-language job control scale that was developed to test Karasek’s (1979) job demands-control model (Richter et al., 2000). Two example items are “I can independently plan and schedule my work tasks” and “I can participate in decisions of my supervisor.” The 5-point response scale ranged from 1 (*disagree*) to 5 (*strongly agree*). In the current study, Cronbach’s alpha for the scale was 0.60, which is somewhat lower than established cut-off values (0.70) and reliability estimates reported in previous validation studies (e.g., 0.73–0.75; Pfaff et al., 2004). However, we deemed a reliability estimate of 0.60 acceptable for a control variable (i.e., not focal construct) with only four relatively heterogeneous items (cf. Gosling et al., 2003).

Finally, we note that the pattern of results was very similar when age, job demands, and job control were not included as control variables in the analyses.

### Statistical Analyses

As our data had a nested structure (i.e., individual employee reports nested within teams), we conducted multilevel modeling with the hierarchical linear modeling (HLM) software to analyze the data (Raudenbush and Bryk, 2002). The employee-level predictors (i.e., age, job tenure, job demands, job control, psychological team health climate) were centered at the group (or team) mean, and the team-level predictors (i.e., team health climate, team size) were centered at the grand (or sample) mean. These centering procedures allowed for unconflated multilevel modeling, which involves controlling for the within-team effects of the aggregated between-team construct (Preacher et al., 2011; Spell et al., 2014). A series of null models (i.e., models without predictors at the employee and team levels) in HLM showed that between 6 and 11% of the variance in our health-related outcomes resided at the team level (**Table 1**). These percentages represent the maximum share of the variance in outcomes that could potentially be explained by employees’ shared perceptions of team health climate.

**Table 1:** Descriptive statistics and correlations of variables.

Variable	*M*	*SD*	ICC^e^	1	2	3	4	5	6	7	8	9	10	11
1. (Psychological) Team health climate^a^	2.30	0.68	0.20	(0.71)	0.09ˆ*									
2. Team size^b^	10.38	5.10	–	–	–									
3. Subjective general health	3.33	0.94	0.07	0.11ˆ***	–	–								
4. Psychosomatic complaints	2.94	0.89	0.08	-0.09ˆ***	–	-0.53ˆ***	(0.81)							
5. Mental health	1.63	0.44	0.07	0.11ˆ***	–	0.47ˆ***	-0.40ˆ***	(0.67)						
6. Work ability	3.78	0.78	0.11	0.15ˆ***	–	0.64ˆ***	-0.56ˆ***	0.53ˆ***	(0.71)					
7. Presenteeism	2.83	0.87	0.06	-0.08ˆ***	–	-0.42ˆ***	0.44ˆ***	-0.33ˆ***	-0.41ˆ***	–				
8. Age^c^	3.44	1.12	–	-0.05ˆ***	–	-0.24ˆ***	0.10ˆ***	-0.08ˆ***	-0.30ˆ***	0.05ˆ***	–			
9. Job tenure^d^	2.22	0.82	–	-0.06ˆ***	–	-0.24ˆ***	0.13ˆ***	-0.09ˆ***	-0.29ˆ***	0.09ˆ***	0.67ˆ***	–		
10. Job demands	2.37	0.81	–	-0.17ˆ***	–	-0.30ˆ***	0.34ˆ***	-0.31ˆ***	-0.47ˆ***	0.28ˆ***	0.24ˆ***	0.30ˆ***	(0.77)	
11. Job control	3.02	0.53	–	0.18ˆ***	–	0.18ˆ***	-0.18ˆ***	0.17ˆ***	0.24ˆ***	-0.12ˆ***	0.01	0.01	0.03ˆ*	(0.60)

*N = 6,449 workers nested in 621 teams. The correlations below the diagonal represent relationships at the individual level (*N* = 6,449 workers); the correlation above the diagonal represents a relationship at the team level (*N* = 621 teams). ^a^Psychological team health climate refers to individual employees’ perceptions of the health climate in their teams (we aggregated psychological team health climate to create our measure of team health climate at the team level, see also **Table 2**). ^b^As team size is a team-level variable, only the correlation with team health climate is reported (above the diagonal). ^c^Age was coded 1 = *20 years or younger*, 2 = *21–30 years*, 3 = *31–40 years*, 4 = *41–50 years*, 5 = *51–60 years*, and 6 = *older than 60 years.*^d^Job tenure was coded 1 = *5 years or less*, 2 = *6–15 years*, and 3 = *16 years or more.*^e^The intraclass correlation coefficient (ICC) is calculated by dividing the between-team variance component (τ_*00*_) by the sum of τ_*00*_ and the within-team variance component (σ^*2*^), and indicates the percentage of between-team variance observed for the variable. Reliability estimates (Cronbach’s α, except for the reliability estimate for mental health, which is a Spearman’s rank-order correlation coefficient), where available, are shown in parentheses on the diagonal. ^∗∗∗^*p <* 0.001; ^∗^*p <0.05**.

To evaluate the factor structure of our multi-item measures (i.e., psychological team health climate, psychosomatic complaints, mental health, work ability, job demands, and job control) and to examine the possibility of common method variance, we conducted confirmatory factor analyses using MPlus version 7 (Muthén and Muthén, 2012). For the two measures with only two items each (i.e., mental health and work ability), factor loadings were constrained to be equal for the purpose of allow model identification. Results showed that a model with six factors fitted the data adequately (χ^2^ = 7115.340, *df* = 194, *p <* 0.001; RMSEA = 0.074; CFI = 0.853; TLI = 0.826; SRMR = 0.078). In contrast, a model with a single factor fitted the data significantly worse (χ^2^ = 23818.867, *df* = 209, *p* < 0.001; RMSEA = 0.132; CFI = 0.500; TLI = 0.448; SRMR = 0.105; Δχ^2^ = 16703.527, *df* = 15, *p* < 0.001). These findings suggest that our multi-item measures are distinct and that it is unlikely that common method bias had an influence on our findings.

## Results

**Table 1** shows the descriptive statistics and employee-level correlations of the study variables (due to the large sample size, the vast majority of correlations are significant at *p* < 0.001). According to Cohen (1988), a correlation coefficient of 0.10 is small, a coefficient of 0.30 is moderate, and a coefficient of 0.50 is large. Accordingly, psychological team health climate was very weakly associated with psychosomatic complaints (*r* = -0.09) and presenteeism (*r* = -0.08), and weakly associated with subjective general health (*r* = 0.11), mental health (*r* = 0.11), work ability (*r* = 0.15), job demands (*r* = -0.17), and job control (*r* = 0.18). Age was very weakly associated with psychological team health climate (*r* = -0.05), mental health (*r* = -0.08), and presenteeism (*r* = 0.05), weakly associated with psychosomatic complaints (*r* = 0.10), subjective general health (*r* = -0.24), and job demands (*r* = 0.24), moderately associated with work ability (*r* = -0.30), and strongly associated with job tenure (*r* = 0.67). The correlations of job tenure with the other study variables were similar. The health-related outcomes were moderately to strongly intercorrelated (see **Table 1**).

**Table 2** shows the results of the multilevel analyses. In terms of effect size, Cohen (1988) suggested that traditional *R*^2^ values of 0.02, 0.13, and 0.26 can be considered small, medium, and large, respectively. In multilevel analyses, only pseudo *R*^2^ values can be computed, which involve the reduction in within- and between-person level variance components (LaHuis et al., 2014). The pseudo *R*^2^ values in **Table 2** indicate that the within- and between-team predictor variables explained 7% of the variance in both mental health and presenteeism, as well as 10% in both subjective general health and psychosomatic complaints. These pseudo *R*^2^ values correspond to small effects sizes. Predictors further explained 23% of the variance in work ability, which indicates a relatively large effect size.

**Table 2:** Results of hierarchical linear modeling analyses predicting health-related outcomes.

	Subjective general health				Psychosomatic complaints				Mental health				Work ability				Presenteeism			
Effect	γ	*SE*	*t*	*p*	γ	*SE*	*t*	*p*	γ	*SE*	*t*	*p*	γ	*SE*	*t*	*p*	γ	*SE*	*t*	*p*
Intercept	3.34	0.02	206.77	<0.001	2.91	0.02	186.48	<0.001	1.63	0.01	219.08	<0.001	3.79	0.01	264.15	<0.001	2.81	0.01	198.09	<0.001
Employee-level predictors																				
Age	-0.13	0.01	-8.86	<0.001	0.03	0.01	2.17	0.030	-0.01	0.01	-1.04	0.297	-0.13	0.01	-12.08	<0.001	-0.02	0.01	-1.68	0.092
Job tenure	-0.07	0.02	-3.62	<0.001	-0.01	0.02	-0.69	0.488	0.01	0.01	0.67	0.501	-0.05	0.01	-3.27	0.001	0.01	0.02	0.75	0.453
Job demands	-0.31	0.02	-18.14	<0.001	0.40	0.02	24.88	<0.001	-0.17	0.01	21.05	<0.001	-0.40	0.01	-32.02	<0.001	0.33	0.02	20.48	<0.001
Job control	0.24	0.02	9.94	<0.001	-0.21	0.02	-9.42	<0.001	0.13	0.01	11.16	<0.001	0.31	0.02	17.61	<0.001	-0.11	0.02	-5.01	<0.001
Psychological team health climate^a^	0.07	0.02	3.45	0.001	-0.05	0.02	-2.93	0.003	0.02	0.01	2.45	0.014	0.06	0.01	4.14	<0.001	-0.04	0.02	-2.13	0.033
Team-level predictors																				
Team size	-0.00	0.00	-1.59	0.111	0.01	0.00	3.89	<0.001	-0.00	0.00	-0.06	0.952	-0.00	0.00	-1.50	0.134	0.01	0.00	3.42	0.001
Team health climate	0.12	0.04	2.76	0.006	-0.04	0.04	-1.04	0.300	0.07	0.02	3.43	0.001	0.16	0.04	4.32	<0.001	-0.10	0.04	-2.73	0.007
Null model τ_00_		0.06				0.06				0.01				0.07				0.04		
Null model σ^2^		0.82				0.73				0.18				0.54				0.71		
Predictor model τ_ 00_		0.07				0.07				0.01				0.08				0.04		
Predictor model σ^2^		0.72				0.64				0.16				0.39				0.65		
Model Pseudo *R*^2^		0.10				0.10				0.07				0.23				0.07		

**N* = 6,449 employees nested in 621 teams. ^a^Psychological team health climate refers to individual employees’ perceptions of the health climate in their teams (we aggregated psychological team health climate to create our measure of team health climate at the team level). Age, job tenure, and psychological team health climate were group mean centered, and team health climate was grand mean centered. Unstandardized coefficients (γ) with standard errors (*SE*) are shown. τ_*00*_ = between-person variance; σ^*2*^ = within-person variance. Model Pseudo *R^*2*^* = ([null model τ_*00*_ + null model σ^**2**^] - [predictor model τ_*00*_ + predictor model σ^*2*^])/(null model τ_*00*_ + null model σ^*2*^).*

At the employee level, psychological team health climate significantly predicted all five health-related outcomes in the expected direction, after controlling for age, job tenure, as well as job demands and job control. Specifically, psychological team health climate positively predicted subjective general health (γ = 0.07, *p* = 0.001), mental health (γ = 0.02, *p* = 0.014), and work ability (γ = 0.06, *p* < 0.001), and negatively predicted psychosomatic complaints (γ = -0.05, *p* = 0.003) and presenteeism (γ = -0.04, *p* = 0.033).

**Table 2** further shows that, at the individual level, age negatively predicted subjective general health (γ = -0.13, *p* < 0.001) and work ability (γ = -0.13, *p* < 0.001), and positively predicted psychosomatic complaints (γ = 0.03, *p* = 0.030). In contrast, age did not significantly predict mental health (γ = -0.01, *p* = 0.297) and presenteeism (γ = -0.02, *p* = 0.092). Job tenure negatively predicted subjective general health (γ = -0.07, *p* < 0.001) and work ability (γ = -0.05, *p* < 0.001). Job tenure did not significantly predict psychosomatic complaints (γ = -0.01, *p* = 0.488), mental health (γ = 0.01, *p* = 0.501), and presenteeism (γ = 0.01, *p* = 0.453). Job demands negatively predicted subjective general health (γ = -0.31, *p* < 0.001), mental health (γ = -0.17, *p* < 0.001), and work ability (γ = -0.40, *p* < 0.001), and positively predicted psychosomatic complaints (γ = 0.40, *p* < 0.001) and presenteeism (γ = 0.33, *p* < 0.001). In contrast, job control positively predicted subjective general health (γ = 0.24, *p* < 0.001), mental health (γ = 0.13, *p* < 0.001), and work ability (γ = 0.31, *p* < 0.001), and negatively predicted psychosomatic complaints (γ = -0.21, *p* < 0.001) and presenteeism (γ = -0.11, *p* < 0.001). As a cross-level predictor, team size was significantly associated with psychosomatic complaints (γ = 0.01, *p* < 0.001) and presenteeism (γ = 0.01, *p* < 0.001), but not significantly associated with subjective general health (γ = -0.00, *p* = 0.111), mental health (γ = -0.00, *p* = 0.952), and work ability (γ = -0.00, *p* = 0.134).

According to our hypotheses, team health climate positively predicts subjective general health (Hypothesis 1), mental health (Hypothesis 2), and work ability (Hypothesis 3), and negatively predicts psychosomatic complaints (Hypothesis 4) and presenteeism (Hypothesis 5), above and beyond the effects of team size, team members’ idiosyncratic perceptions of health climate, and the other individual-level control variables. The results in **Table 2** show that Hypotheses 1, 2, 3, and 5 were supported, whereas Hypothesis 4 was not supported. Specifically, team health climate positively predicted subjective general health (γ = 0.12, *p* = 0.006), mental health (γ = 0.07, *p* = 0.001), and work ability (γ = 0.16, *p <* 0.001), and negatively predicted presenteeism (γ = -0.10, *p* = 0.007). In contrast, team health climate did not significantly predict psychosomatic complaints above and beyond the control variables (γ = -0.04, *p* = 0.300).

### Additional Analyses

**FIGURE 1:**
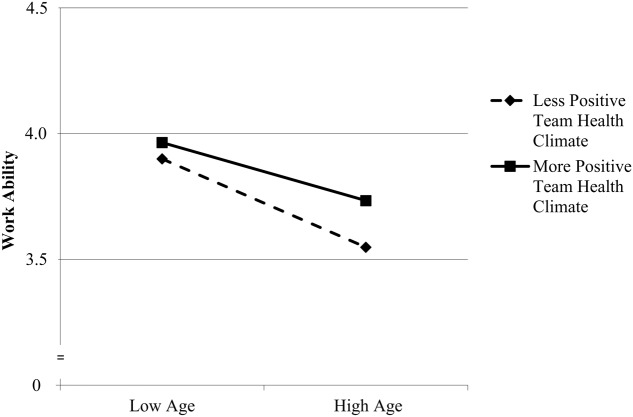
**Relationship between age and work ability moderated by team health climate**.

We conducted a series of additional analyses in which not only the main effects of team health climate, but also the cross-level moderating effects of team health climate on the relationships between employee age- and health-related outcomes were tested. Results showed that only the employee-level relationship between age and work ability was moderated by team health climate (interaction effect: γ = 0.07, *SE* = 0.02, *t* = 3.01, *p* = 0.003). Simple slope analyses showed that the relationship was stronger negative for employees in teams with a less positive team health climate (-1 *SD*: γ = -0.16, *SE* = 0.01, *t* = -11.01, *p* < 0.001) compared to employees in teams with a more positive team health climate (+1 *SD*; γ = -0.10, *SE* = 0.01, *t* = -7.61, *p* < 0.001). This cross-level interaction effect is shown in **Figure 1**. The finding suggests that a positive team health climate buffers the negative relationship between employee age and work ability, but it does not seem to impact on the relationships between age and the other health-related outcomes.

## Discussion

Maintaining and improving individual employee health is not only important with regard to employees’ quality of life, but also with regard to increased productivity and reduced costs at the organizational and societal levels (Danna and Griffin, 1999). On average, a German white-collar worker was 12 days on sickness leave in the year 2014, which corresponds to costs of Euro 1,519 per worker per year estimated based on average worker salary (2% of Germany’s gross domestic product) and of Euro 2,378 per worker per year estimated based on lost productivity (3.1% of gross domestic product; Bundesanstalt für Arbeitsschutz und Arbeitsmedizin, 2016). Even though organizational psychologists suggested already more than two decades ago that workplace climate predicts health-related outcomes (Ilgen, 1990; Stokols, 1992), very little empirical research has so far been conducted to demonstrate this link. The few studies on health climate that exist are limited due to a small number of higher level units (i.e., teams, worksites, or organizations), lack of evidence for within-unit agreement of employees in their health climate perceptions, the absence of multilevel analyses of hierarchically structured data, or the non-assessment of relevant health-related outcomes (Ribisl and Reischl, 1993; Basen-Engquist et al., 1998; Ernsting et al., 2013; Sonnentag and Pundt, 2013, 2016; Zweber et al., 2016).

The goal of this study, therefore, was to contribute to this research domain by examining the relationships between team health climate and health-related outcomes using a large sample of 6,449 employees in 621 teams. We hypothesized and found that team health climate as a collective team property was positively related to employees’ subjective general health, mental health, and work ability, and negatively related to presenteeism, above and beyond the effects of team size, age, tenure, job demands, job control, and individual employees’ individual perceptions of team health climate. Thus, our findings are consistent with assumptions based on climate theory (Schneider et al., 2013), signaling theory (Connelly et al., 2011), and social exchange theory (Blau, 1964), as well as previous studies suggest that health climate is a contextual resource that facilitates health-related outcomes among employees.

Contrary to our expectation, we did not find a significant relationship between team health climate and psychosomatic complaints in this study. A possible explanation for this finding may be that the causes that lead to the development of rather objective (and possibly more strongly genetically determined) physical symptoms such as headaches, backaches, neck and shoulder pain, and tiredness are less likely to be influenced by environmental factors such as team health climate, and more likely to be influenced by idiosyncratic medical conditions (Watson and Pennebaker, 1991). In contrast, more subjective and behavioral outcomes such as subjective general health, mental health, work ability, and presenteeism may be more susceptible to the influence of the team environment and team health climate in particular.

Interestingly, psychological team health climate was significantly associated with all five health-related outcomes. Due to the cross-sectional design of our study, it remains unclear, however, whether individual employees’ perceptions of their team health climate influence outcomes or, alternatively, whether employees attribute their health-related outcomes, at least in part, to their team environment. In a similar vein, it may be possible that the health-related outcomes of employees within a team influenced team health climate in a bottom-up manner, and not vice versa, as we assumed, in a top-down manner.

Employee age and job tenure were negatively associated with subjective general health and work ability, and age was positively associated with psychosomatic complaints. These findings contradict results of a recent meta-analysis on age and health by Ng and Feldman (2013). These authors found that age was unrelated to subjective general health and psychosomatic complaints; work ability was not included in the meta-analysis but longitudinal research has shown that work ability declines with age (Ilmarinen et al., 1997). It is important to point out here that there were only few older employees represented in Ng and Feldman’s (2013) meta-analysis, with the oldest employees being 58 years old, and thus range restriction may have attenuated the relationships between age and health outcomes. In support of this assumption, and consistent with our current findings, Stephan et al. (2012) showed that age was negatively related to subjective general health when older adults are included in the sample.

Finally, additional analyses showed that a positive team health climate weakened the negative relationship between age and work ability, whereas team health climate did not impact on the other relationships between age- and health-related outcomes. Thus, older employees appear to benefit more from a positive team health climate than younger employees in terms of work ability. Interestingly, of all outcomes in this study, work ability had the strongest negative relationship with employee age, and also the strongest relationship with team health climate. It may be possible that team health climate is particularly important for older employees’ work ability because the team context may offer opportunities for the use of compensation strategies (e.g., asking others for help) when employees’ capabilities do not mesh well with their physical and mental job demands (Weigl et al., 2013). More broadly, the interactive effect of age and team health climate on work ability suggests that team health climate may be a contextual resource for successful aging in the work context (Zacher, 2015).

### Strengths, Limitations, and Future Research

This study has several strengths and limitations. The large sample and multilevel design constitute clear strengths, as does the assessment of a range of health-related outcomes that fall within the broad domains of physical and mental health as well as behavioral indicators of health. However, the study is also limited in that the cross-sectional, correlational design does not allow inferences about causality. Future research should therefore examine the effects of team health climate on changes in health-related outcomes over several months or years, or conduct quasi-experimental intervention studies or randomized control trials in which team health climate is manipulated (cf. Basen-Engquist et al., 1998). Also, the data we collected for this study did not allow us to differentiate different work areas and to compare them, which might be worthwhile to do in future research.

Second, all data collected in this study was self-reported by employees using an online questionnaire. Thus, it may be possible that our findings were biased by common method variance and socially desirable responding. By conducting confirmatory factor analyses and by aggregating individual employees’ ratings of health climate to the team level and by using multilevel analyses to regress individual employee outcomes on team health climate while controlling for psychological team health climate, we were able to partially address concerns about common method bias. However, future studies should attempt to collect health-related outcomes from multiple sources, including supervisors and peers, and by obtaining objective employee outcomes such as sickness absences or medical diagnoses. We attempted to reduce socially desirable responding by ensuring complete anonymity and confidentiality to participants. Inspection of the scale means and standard deviations suggested that ratings were not attenuated or inflated; however, we cannot complete rule out the possibility that participants’ responses were somehow biased.

A third potential limitation concerns the length of the measures used in this study. Due to time constraints, we had to use short and rather global measures. We used a three-item measure of general team health climate that was adapted from previous research (Basen-Engquist et al., 1998; Sonnentag and Pundt, 2013) and had acceptable reliability and aggregation statistics in this study. Moreover, we provided evidence for the convergent validity of our measure by showing a strong positive relationship with a previously used general health climate scale (Basen-Engquist et al., 1998). However, future research could assess additional, more specific dimensions of team health climate, for instance, supervisor and coworker support for employee health (Ribisl and Reischl, 1993; Zweber et al., 2016), smoking norms (Basen-Engquist et al., 1998), and eating and exercise climates (Sonnentag and Pundt, 2016). While we expected that general team health climate would predict relatively broad health-related outcomes, it may be that these more specific health climate dimensions better predict specific employee health behaviors and outcomes.

Fourth, the use of single-item measures of subjective general health and presenteeism in the current study may be criticized, as such measures do not allow estimating internal consistency reliability. However, subjective general health and presenteeism were moderately correlated with the other health-related outcomes in the present study, and previous research has demonstrated their reliability and validity (Lundberg and Manderbacka, 1996; Aronsson et al., 2000; Pinquart, 2001; DeSalvo et al., 2006b; Demerouti et al., 2009). Moreover, researchers have suggested that relatively narrow and unambiguous constructs such as general health, presenteeism, and mental health can be assessed with a single item (Wanous et al., 1997; Fisher et al., 2016). Nevertheless, future studies in which employees have more time available to complete surveys should use longer scales which allow estimating internal consistency reliability and which may represent multiple dimensions of a construct. Similarly, the reliability estimate for the job control scale used in this study was somewhat below the conventional cut-off of 0.70. We recommend that researchers use more homogeneous short scales to measure job control (e.g., Morgeson and Humphrey, 2006).

Finally, participants in this study came from a single organization in the health insurance industry. It may be argued that health is a priority for all teams in a health insurance company. However, health-related topics are not necessarily part of meeting discussions in this company. Instead, team members discuss backlogs, service levels, and efficiency issues (similar to a call center). Moreover, our findings suggested that teams within the organization differed significantly in their team health climate, despite a shared organizational level human resource management (indeed, it constitutes a strength of this study that these background variables were held constant). Nevertheless, we acknowledge that it may not be possible to readily generalize the findings of our study with white-collar workers to blue-collar workers in industries such as construction and manufacturing. Future research should therefore collect data on team health climate from more diverse occupational samples to support the external validity of the results.

### Theoretical and Practical Implications

Our findings have a number of implications for future theory development and occupational health management practice. Researchers could develop a conceptual framework, based on the broader organizational climate literature (Schneider et al., 2013), that outlines the company-, team-, and employee-level antecedents and consequences of team health climate. For instance, a positive team health climate may be easier to establish in certain industries (e.g., health care, food) than in others (e.g., construction, entertainment). Moreover, employee attitudes and behaviors may be more difficult to change through organizational interventions in certain industries, thus more individual-based interventions may be needed (e.g., Ernsting et al., 2013; Lippke et al., 2015). The framework should also distinguish between more immediate consequences (or mediators of the effects) of team health climate (e.g., behavior and acute health-related outcomes such as irritation) and more distal outcomes (e.g., chronic health outcomes such as burnout). This conceptual framework could also integrate ideas from the literature on personal and contextual resources (Hobfoll, 2001), job demands and resources (Demerouti et al., 2001), dynamic person-team fit (Zacher et al., 2014), as well as goal selection, optimization, and compensation mechanisms that enhance favorable health-related outcomes (Müller et al., 2013; Weigl et al., 2013).

The pseudo *R*^2^s obtained in this study suggested that psychological and shared team health climates, together with the control variables, explained only between 7 and 23% of the total variance in the health-related outcomes. An explanation for these results is that team health climate constitutes a rather distal predictor and only one of many factors that may impact on health-related outcomes. For instance, individual-level factors such as genetics and personality dispositions, as well as more proximal situational factors such as leadership behavior also influence health-related outcomes (Watson and Pennebaker, 1991; Montano et al., 2016). Nevertheless, it is possible that team health climate has stronger effects on some teams and among certain groups of employees than others. Thus, future theorizing should also consider potential team- and employee-level moderators of the effects of team health climate. In this study, we found that team health climate had particularly positive effects on older employees’ work ability. While this finding is consistent with conservation of resources theory applied to age-related resource losses (Hobfoll and Wells, 1998), it remains a question for future research why team health climate did not moderate the relationships between employee age and the other health-related outcomes in this study (many of which were also related to age).

In terms of practical implications, team health climate needs to be taken into account in health interventions because our results suggest that employees’ shared perceptions of the extent to which their team is concerned, cares, and communicates about health issues are positively related to subjective general health, mental health, and work ability, and negatively related to presenteeism. These employee outcomes have been shown to be associated with significant long-term consequences such as individuals’ quality of life, mortality, onset of retirement, absenteeism, and company productivity and costs (De Boer et al., 2002; Goetzel et al., 2004; DeSalvo et al., 2006a; Sell, 2009). As absenteeism, productivity loss, and increased health care and insurance costs due to ill-health are very costly for organizations and society (Danna and Griffin, 1999), improving team health climate is an important endeavor.

Human resource managers and supervisor could encourage team members to discuss health issues and provide teams with health-related information and practical support (e.g., physical and mental health workshops, employee assistance programs). Moreover, managers and supervisors can gain a more differentiated picture of employee perceptions of how the team supports positive health outcomes and identify areas where improvements are needed. Recent research suggests that supervisors and team leaders may be important role models in terms of health-related outcomes (Koch and Binnewies, 2015). The finding of a moderating effect of team health climate on the negative relationship between age and work ability has implication for managing the aging workforce. Practitioners interested in maintaining older employees’ work ability, as well as subsequent outcomes such as quality of life and delayed retirement onset, should ensure that older employees have access to health-related information and discussions within the team.

## Conclusion

In summary, this study contributes to the occupational health psychology literature by extending research on the topic of health climate, and by showing that general team health climate was related to several important health-related outcomes, above and beyond individual employees’ idiosyncratic perceptions of team health climate, in a large sample of white-collar workers. However, some aspects could not be analyzed with the current data; for instance, it was not possible to differentiate different work areas and compare them, which might be worthwhile to do in future research. Moreover, future research is now needed that examines multiple dimensions and additional outcomes of team health climate, health climate at the organizational level, the mediating mechanisms and boundary conditions of relationships between health climate and employee health-related outcomes, and the effects of health climate in different groups of employees and in different types of occupations. This line of research on health climate has the potential to contribute importantly to the improvement and maintenance of employee health and thus individuals’ quality of life, as well as to increased productivity and reduced health care and insurance costs for organizations and society as a whole.

## Author Contributions

HS conducted the study, HS drafted the first version of the manuscript, HZ and SL provided feedback, and all authors edited and revised the manuscript.

## Conflict of Interest Statement

The authors declare that the research was conducted in the absence of any commercial or financial relationships that could be construed as a potential conflict of interest.
